# Current Role of Electrocardiographic Imaging in Patient Selection for Cardiac Resynchronization Therapy

**DOI:** 10.3390/jcdd11010024

**Published:** 2024-01-15

**Authors:** Saer Abu-Alrub, Marc Strik, Peter Huntjens, Michel Haïssaguerre, Romain Eschalier, Pierre Bordachar, Sylvain Ploux

**Affiliations:** 1 Cardiology Department, Centre Hospitalier Universitaire Clermont-Ferrand, 63000 Clermont-Ferrand, France; reschalier@chu-clermontferrand.fr; 2 Cardio-Thoracic Unit, Bordeaux University Hospital (Centre Hospitalier Universitaire), 33600 Pessac-Bordeaux, France; marcstrik@gmail.com (M.S.); sylvain.ploux@gmail.com (S.P.); bordacharp@hotmail.com (P.B.); jean-michel.haissaguerre@chu-bordeaux.fr (M.H.); 3 IHU Liryc, Electrophysiology and Heart Modeling Institute, Fondation Bordeaux Université, F-33600 Pessac-Bordeaux, France; 4 Division of Cardiology, Washington University in St. Louis, St. Louis, MO 63110, USA; p.huntjens@wustl.edu

**Keywords:** mapping, imaging, CRT, ECGi, belt

## Abstract

Cardiac resynchronization therapy (CRT) is a recognized therapy for heart failure with altered ejection fraction and abnormal left ventricular activation time. Since the introduction of the therapy, a 30% rate of non-responders is observed and unchanged. The 12-lead ECG remains the only recommended tool for patient selection to CRT. The 12-lead ECG is, however, limited in its inability to provide a precise pattern of regional electrical activity. Electrocardiographic imaging (ECGi) provides a non-invasive detailed mapping of cardiac activation and therefore appears as a promising tool for CRT candidates. The non-invasive ventricular activation maps acquired by ECGi have been primarily explored for the diagnosis and guidance of therapy in patients with atrial or ventricular tachyarrhythmia. However, the accuracy of the system in this field is lacking and needs further improvement before considering a clinical application. On the other hand, its use for patient selection for CRT is encouraging. In this review, we introduce the technical considerations and we describe how ECGi can precisely characterize ventricular activation, especially in patients with left bundle branch block, thus identifying the electrical substrate responsive to CRT.

## 1. Introduction

Cardiac resynchronization therapy (CRT) improves the quality of life, left ventricle (LV) ejection fraction and survival in patients with heart failure with reduced ejection fraction (HFrEF) and wide QRS [1]. However, the non-response rate of approximately 30% remained unchanged since the introduction of the therapy. The most recent European guidelines for CRT implantation recommend to rely on the 12-lead ECG for the selection of CRT candidates, favoring a QRS of at least 130 ms and a left bundle branch block (LBBB) pattern [2]. Improving patient selection for CRT has become a major priority, vectorcardiography showed a better accuracy to CRT response than a QRS duration/pattern but also relies on the 12-lead ECG, and has yet to become standard practice [3]. Intracardiac mappings are widely used for the treatment and comprehension of tachyarrhythmia; however, its application in CRT guidance is anecdotical due to its invasive nature. Electrocardiographic imaging (ECGi) allows for a detailed mapping of the ventricular activation and automated parameters exclude intra- and inter-observer variability [4,5]. Therefore, ECGi appears promising to identify profile of ventricular dyssynchrony favorable for the CRT response. 

In this review, we will detail the technical aspects of ECG and discuss its potential place in patients’ selection for CRT.

## 2. Electrocardiographic Imaging: Technical Aspects

### 2.1. Acquisition Method

The details of the ECGi system have been previously described [6]. The ECGi system employs a single-use vest equipped with more than 250 electrodes to record electrical potentials from the body’s surface. It uses these signals to calculate how the various heart regions are activated by solving the inverse problem [7]. Each of these vest electrodes contains a marker that can be visualized using a CT scan, as the software requires information about the shapes of both the heart and the torso surfaces. The patient undergoes a thoracic non-contrast-gated CT scan, which provides information about the epicardial geometry and the positions of the torso electrodes in a consistent reference frame. Subsequently, the collected body surface potentials and computed tomography images are merged and processed to reconstruct over 2500 epicardial unipolar electrograms. These electrograms enable the continuous creation of isochrones, allowing for beat-by-beat analysis. Local ventricular activation times are determined as the duration between the onset of the QRS complex or the pacing artifact and the point of maximum negative slope on each unipolar electrogram. These activation times are then plotted onto the ventricular surface, ultimately generating a three-dimensional activation map. 

The ECG belt is an innovative surface mapping system composed of a two-piece electrode array that wraps around the upper torso. It consists of a 53-electrode body surface mapping system (adapted from Heartscape Technologies, Verathon, Seattle, WA, USA) with a multichannel amplifier and customized software for data recording. This specialized software then converts the multi-electrode ECG data into color-coded isochronal maps, which are presented in both anterior and posterior views. Two metrics of electrical heterogeneity can be calculated using this system: the standard deviation of activation times from all electrodes, which provides a global measure of electrical heterogeneity, and left thorax activation times, which averages the activation times of electrodes on both the anterior and posterior surfaces of the left thorax (located to the left of the sternum on the anterior and left of the spine on the posterior surfaces). The data collected by the ECG belt is processed offline to generate measurements of electrical heterogeneity and color-coded isochronal maps. One significant advantage of this system over ECGi is that it does not require a CT scan. This simplifies the workflow, eliminates radiation exposure, but reduces the resolution and accuracy since it does not integrate patient-specific cardiothoracic anatomy into the analysis.

Electrical dyssynchrony parameters derived from ECGi are multiple. The total activation time (TAT) defined as the duration (in milliseconds) from the earliest to the latest site of ventricular activation. The ventricular electrical uncoupling (VEU) calculated as the difference between the mean LV and RV activation times (in milliseconds); a positive value reflects RV pre-activation, whereas a negative value reflects LV pre-activation. The activation delay vector (ADV) describes the imbalance of ventricular depolarization, or activation delay, in time (by its amplitude) and in space by its direction. ADV was calculated by combining the depolarization times and coordinates (X, Y and Z distances from the center of the heart in millimeters) of all virtual electrodes.

Electrical dyssynchrony parameters derived from ECG belt include standard deviation of activation times (SDAT) and left total activation time (LTAT), which averages activation times from electrodes on the left side of the thorax.

### 2.2. Limitations

The ECGi system underwent validation through experimental and patient-based assessments, encompassing various physiological and pathological conditions. This validation involved comparisons with direct epicardial mapping during open-heart surgery and catheter intracardiac mapping [7]. Nonetheless, a validation study revealed a significant lack of agreement between ECGi activation mapping and contact mapping. Notably, the correlation between these two mapping methods was strong for wide QRS patterns, such as those seen during asynchronous ventricular activation in candidates for cardiac resynchronization therapy (CRT) [8]. Since ECGi technology relies on local ventricular activation, electrically inactive regions in patients with previous myocardial infarctions can introduce inaccuracies into the activation map.

## 3. Impact of Electrical Asynchronism on Myocardial Contraction

The left bundle represents the main bundle of the conduction system, and has been extensively studied in animal models, particularly in dogs, whose conduction system is similar to that of humans [9]. The presence of LBBB produces a typical activation map, with an early activation of the apicolateral wall of the RV and late activation of the lateral wall of the LV [10]. This interventricular uncoupling causes an asynchronous contraction between the RV, the septal wall of the LV and the lateral wall of the LV. A left bundle branch block thus represents the reference model for electrical asynchronism; moreover, by extension, any lesion in the conduction pathways is likely to result in electrical asynchronism.

**Figure 1:**
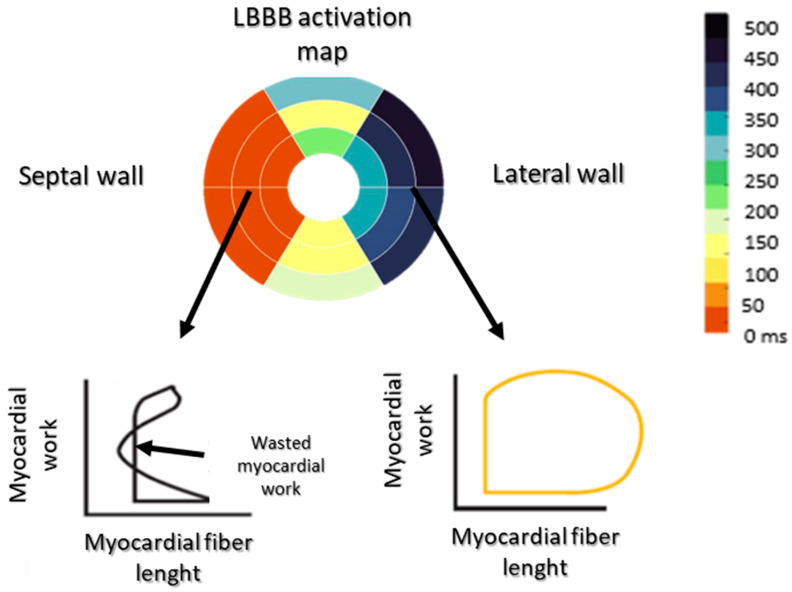
Representation of the left ventricular myocardial work in LBBB. During LBBB, the septal wall contracts in a low-pressure cavity without encountering any resistance; the myocardial work is therefore wasted. The lateral wall undergoes an initial stretch in response to the contraction of the septal wall, and then, through the Frank–Starling effect, develops a greater contractile force/work than in the absence of electrical asynchrony.

Myocardial depolarization initiates a contraction; thus, in the LBBB, beyond the temporal asynchrony of the early and late activated walls, their contractile mode is diametrically opposed. On the one hand, the septal wall, which is the first to be activated, will contract in a low-pressure cavity without encountering any resistance; on the other hand, the lateral wall of the LV will undergo an initial stretch in response to the contraction of the septal wall. Then, through the Frank–Starling effect, it will develop a greater contractile force than in the absence of electrical asynchronism (Figure 1). The myocardial workload of the septal wall is therefore low, while that of the lateral wall is increased. This heterogeneous workload distribution within the LV generates cellular changes in favor of hypertrophic remodeling of the lateral wall, associated with energetic and electrical changes [11,12].

Electrical asynchrony and its effects on myocardial contraction ultimately have an impact on left ventricular systolic and diastolic function. Charging conditions have a direct impact on myocardial contraction and relaxation. To study the isolated effect of electrical asynchrony on systolic and diastolic function, it is essential to control charging conditions so as to vary only the sequence of electrical activation. In this way, it has been demonstrated that, under equal load conditions, the presence of electrical asynchrony (by right ventricular pacing) reduces LV systolic function, increases LV end-diastolic volume and pressure, and results in a “rightward” shift of the pressure–volume curve [13,14].

**Figure 2:**
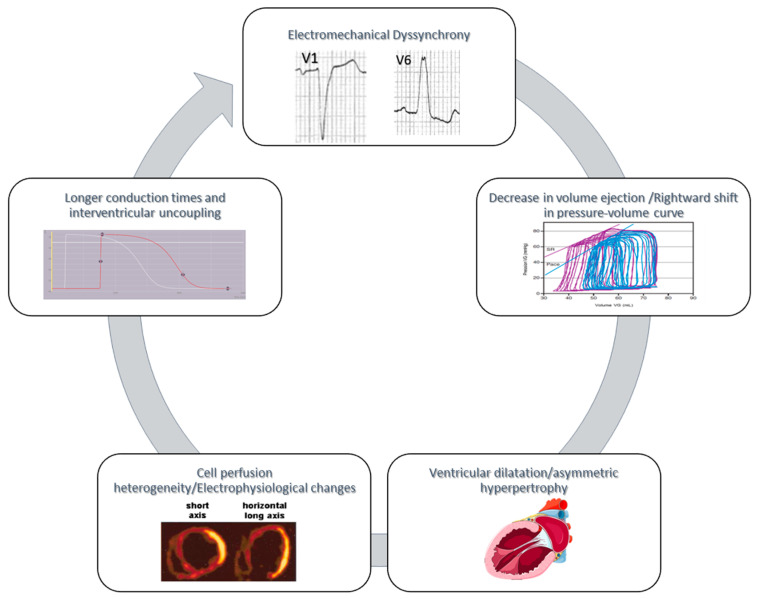
Impact of the electromechanical asynchronism on systolic dysfunction. Electrical dyssynchrony increases end-diastolic volumes and dilates the ventricular cavity, shifting the pressure–volume loop to the right. The latest wall activated develops a greater myocardial workload, resulting in the activation of hypertrophy pathways associated with electrophysiological remodeling and action potential lengthening. These structural changes favor longer electrical impulse propagations and aggravate electromechanical dyssynchrony.

The presence of a left bundle branch block therefore has a direct impact on myocardial contractility and (more generally) on systolic function. The decrease in systolic function induced by electrical asynchrony will increase end-diastolic volumes and ultimately dilate the ventricular cavity, shifting the pressure–volume loop to the right. In addition, the wall that is activated the latest develops a greater myocardial workload, resulting in the activation of hypertrophy pathways associated with electrophysiological remodeling and action potential lengthening. These structural changes favor longer electrical impulse propagations and aggravate electromechanical asynchrony (Figure 2).

## 4. Role of Electrocardiographic Imaging in Patient Selection

### 4.1. Electrocardiographic Imaging Allows a Better Understanding of Ventricular Activation

A major limitation of the 12-lead ECG is its inability to provide a precise pattern of regional electrical activity. It has been observed that patients with LBBB benefit the most from CRT. Moreover, the definition of LBBB remains heterogeneous between studies with considerable intra- and inter-observer variability [15]. These different LBBB definition results in a divergent clinical impact [16]. 

In comparison to the 12D ECG, ECGi provides a more detailed mapping of ventricular activation. In patients with left bundle branch block (LBBB), the sequence of ventricular activation tends to have limited variability between individuals, making the maps easily distinguishable. Our observations revealed that in patients with LBBB, there is a right ventricular (RV) breakthrough with a rapid and outward spread of activation along the RV free wall. This is followed by activation spreading circumferentially toward the left ventricle (LV) [17,18].

**Figure 3:**
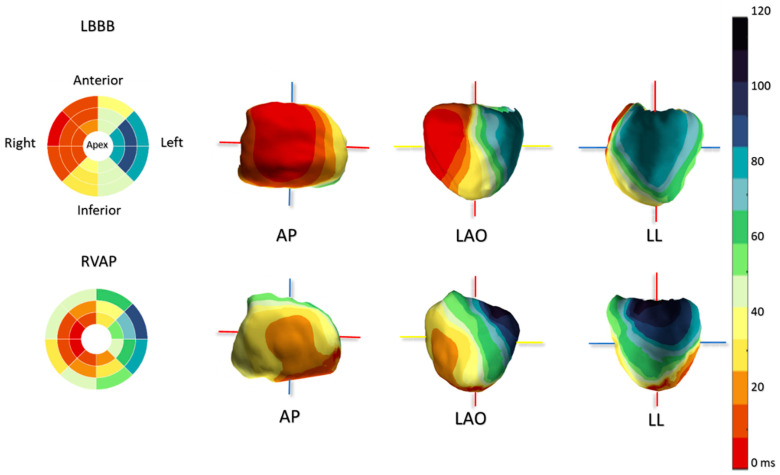
Typical 3D activation map of LBBB and RVAP. Averaged activation map per segment of 16 patients with normal axis LBBB (**left top** panel) and of 13 RVAP patients (**left bottom** panel). Three-dimensional activation maps of a typical LBBB (**right top** panel) and RVAP (**right bottom** panel). LBBB: left bundle branch block; RVAP: right ventricular apical pacing.

On a standard 12-lead ECG, LBBB and right ventricular apex pacing (RVAP) often appear to be quite similar. However, in an ECGi study involving 24 patients with heart failure and reduced ejection fraction (HFrEF), we compared ventricular activation patterns during LBBB and RVAP and identified significant differences. Patients undergoing RV pacing exhibited a distinct activation pattern characterized by an apical RV breakthrough followed by activation at the RV base. During RVAP, the RV activation was notably prolonged compared to LBBB. Additionally, the earliest site of activation in the LV consistently appeared at the apex, followed by activation propagating from apex to base (Figure 3) [19].

Compared to patients with LBBB, patients with NICD demonstrated heterogeneous patterns of activation and variable extent of activation delay. However, the direction of the activation delay vector was consistently directed toward the LV lateral wall.

Vectorcardiography (VCG) consists of three orthogonal leads, X, Y and Z, which together form a 3D vector loop that is derived from the 12-lead ECG. The Kors regression method is the reference method for VCG calculation. The QRS area derived from it is correlated with a positive response to CRT [3]. A strong interventricular dyssynchrony, as in LBBB, will result in a vectorial force strongly oriented toward the LV that will result in a bigger QRS area on the VCG. ECGi also allows to measure the magnitude and direction of the main vectorial force by the ADV, as the VCG it includes amplitude and direction (X, Y and Z distances from the center of the heart in millimeters). Strik et al. demonstrated that ADV magnitude was larger in LBBB (117 ± 25 ms) than in NICD (70 ± 29 ms, *p* < 0.05), and ADV accurately predicted the acute (AUC = 0.93) and chronic (AUC = 0.90) response to CRT [20], while the QRS area had only an AUC of 0.69 [21].

### 4.2. Electrical Dyssynchrony and Interventricular Dyssynchrony Are the Main Parameters for CRT Response

Using ECGi, Ploux et al. addressed the electrical consequences of biventricular pacing (BVP) combined with invasive hemodynamic measurements before and after BVP in a population of patients with heart failure (n = 61) covering a variety of conduction patterns (13 narrow QRS, 22 NICD and 26 LBBB) [22]. They observed that BVP does not fully reverse the conduction impairment, but interestingly induces a similar degree of electrical dyssynchrony, independently of baseline electrical dyssynchrony. Thus, since BVP results in similar ventricular activation regardless of the baseline conduction characteristics, improvement or worsening in function are mostly determined by the severity of the ventricular conduction impairment during baseline conduction, which also determines the extent to which dyssynchrony is corrected. ECGi parameters were a better predictor of a clinical response at 6 months when assessed in baseline intrinsic conduction rather than in BVP, or when using the degree of change from baseline (AUC 0.90 versus 0.53 and 0.76, respectively) [20]. Therefore, the hemodynamic response to CRT is highly contingent on the amount of baseline electrical dyssynchrony, and CRT may induce iatrogenic dyssynchronopathy in patients with insufficient electrical dyssynchrony at baseline [22]. 

Currently, the assessment of electrical dyssynchrony relies on surface ECG parameters like QRS duration and its morphology. This remains the parameter of choice in the 2021 ESC guidelines, and it has remained unchanged since the 2013 guidelines. However, ECGi can offer a better prediction of the hemodynamic and long-term clinical response to cardiac resynchronization therapy (CRT) by identifying interventricular dyssynchrony.

Ploux et al. conducted a study to investigate the utility of ECGi for predicting the CRT response in 33 consecutive CRT candidates, comprising 18 patients with left bundle branch block (LBBB) and 15 patients with non-ischemic cardiomyopathy (NICD) [17]. Electrocardiographic maps generated via ECGi revealed consistent patterns of activation with greater ventricular electrical uncoupling (VEU) and left ventricular total activation time (LVTAT) in patients with LBBB compared to patients with NICD, who exhibited more diverse activation patterns and shorter VEU and LVTAT. Using a cutoff value of 50 milliseconds for VEU, ECGi accurately identified CRT responders with a sensitivity of 90% and specificity of 82%, irrespective of the presence of LBBB. Patients with a VEU exceeding 50 ms had a 42-fold higher likelihood of responding to CRT (*p* < 0.001). Interestingly, significant VEU was identified in all patients with LBBB, potentially explaining the high response rate to CRT in this subgroup. Notably, some patients with NICD displayed ventricular activation patterns resembling LBBB (20 to 50%), accompanied by a substantial VEU detectable with ECGi [17,23]. Recognizing significant VEU in patients with prolonged QRS duration on surface ECG but lacking typical LBBB morphology may represent a promising strategy for identifying non-LBBB patients who could benefit from CRT.

## 5. CRT Optimization Using Electrocardiographic Imaging

### 5.1. Correction of Dyssynchrony in Non-Responders and Responders

Many recent studies used ECGi and the ECG belt to better understand biventricular activation during CRT and how it could be optimized. Waddingham et al. explored the level of dyssynchrony during BVP with and without algorithms for optimized CRT. The dynamic algorithm, SyncAV (Abbott, Sylmar, CA, USA), continually programs the AV delay shorter than the intrinsic PR interval by a customizable offset (either fixed or percent of PR interval) to synchronize the paced ventricular activation wavefronts and achieve fusion with intrinsic conduction. Sync AV can be combined with Multipoint Pacing. This study was conducted with ECGi after CRT implantation, and only patients with LBBB were included (n = 25). Electrical parameters were measured during intrinsic conduction and the following pacing modes: BVP with the nominal AVD (BiV, static paced/sensed AVD of 140/110 ms), LV-only single-site pacing with SyncAV (LVSS + SyncAV), BVP with SyncAV (BiV + SyncAV), LV-only MPP with SyncAV (LVMPP + SyncAV), and biventricular MPP with SyncAV (MPP + SyncAV). The MPP + Sync AV provided the best parameters in most patients with a greater reduction of QRS duration from baseline by 27.2% [121 (IQR: 108–127) ms]; LVAT by 27.4% [90 (IQR: 83–98) ms]; and VEU by 55.9% [21 (IQR: 11–27) ms]. This study confirms that acute fusion optimization can be successfully and reliably achieved with the use of a device algorithm. However, this study is lacking clinical data as the levels of responses to CRT were not studied; therefore, the clinical benefit of these electrical optimizations are unknown. Moreover, MPP has failed to demonstrate strong clinical outcomes compared to conventional CRT, with no effect on left ventricular end-systolic volume (LVESV) (mean difference, 0.39; 95% CI, −11.12 to 11.89; *p* = 0.94), and no significant difference regarding hospitalization for HF was found (odds ratio, 0.70; 95% CI, 0.32 to 1.54; *p* = 0.38) [24]. This lack of clinical benefits of MPP was very recently confirmed in a randomized controlled study in the MORE-CRT MPP trial. This trial included non-echocardiographic responders to CRT at 6 months of BiVP; 541 patients then received MPP, and 570 patients continued BiVP for an additional 6 months. At the end of follow-up, 29.4% of patients in the MPP group vs. 30.4% in the BiVP group became responders (*p* = 0.743). This study pointed out that some patients have a delayed or slowly incremental CRT benefit, disregarding the resynchronization method [25]. Overall, patient selection is the main determinant of the responders’ rates rather than dyssynchrony optimization.

Within the responders’ population, dyssynchrony optimization seems to be the main determinant for resynchronization optimization. Elliott et al., using ECGi, demonstrated with a sample of 10 patients that LBB pacing (LBBAP) and endocardial LV pacing reached improved resynchronization parameters when compared with classic BiV pacing. During baseline rhythm, mean LVAT was 105.2 ± 22.8 ms. Compared to baseline, LVAT was significantly shorter during BiV-epi (79.2 ± 13.1 ms; *p* = 0.006) with additional shortening achieved with both BiV-endo (48.5 ± 14.9 ms; *p* <0.001) and LBBAP (48.9 ± 12.5 ms; *p* < 0.001) [26]. The electrophysiological results are in line with clinical observations. Indeed, in a randomized prospective study, the LBBP-RESYNC study, 40 patients with non-ischemic cardiomyopathy and LBBB were resynchronized either with LBBAP or classic BiV pacing. After 6 months of follow-up, the LVEF improvement was better in the LBBAP group (mean difference: 5.6%; 95% CI: 0.3–10.9; *p* = 0.039); they also had greater reductions in left ventricular end-systolic volume (−24.97 mL; 95% CI: −49.58 to −0.36 mL) and NT-proBNP (−1071.80 pg/mL; 95% CI: −2099.40 to −44.20 pg/mL). However, the rate of responders was the same in both groups based on the LVESV reduction ≥15% (LBBAP: 94.7% vs. Classic BiV pacing: 85.7%) [27].

### 5.2. Place of ECGi in LV Lead Placement

Parreira et al. used ECGi to determine the relation between the latest electrically activated site (LVEAS) in the LV with the proximity of LV pacing site (LVPS). One hundred and eleven patients with LBBB and an indication for CRT were included between March 2019 and May 2021. Non-invasive 3D electrical activation mapping was undertaken at a single study visit occurring 6–24 months post-implantation. Moreover, pacing was deactivated to allow for the acquisition of intrinsic conduction, and the CT scan was used to assess the LV lead position. All patients had clinical FU visits between 6 and 12 months of post-implant, including echocardiographic assessment. Cardiac resynchronization therapy non-response was defined as a left ventricular end-systolic volume (LVESV) reduction of <15%. The main result of this study showed that a distance of >47 mm between the LVEAS and the LVPS divided the responder and non-responder groups to provide the best balance between sensitivity and specificity (sensitivity 87%, specificity 92%, positive predictive value 84% and negative predictive value 93%) and an AUC of 0.931 [28]. These results are very encouraging; however, it should be kept in mind that the authors did not study the activation parameters during BVP. Thus, it could not be concluded on whether responders had better activation than non-responders during BVP. It would have been interesting to explore the outcome of non-responders after repositioning the LV lead in a better position.

Nguyên et al. demonstrated the feasibility of a non-invasive preimplantation mapping integrating coronary venous anatomy, myocardial scar localization and ECGi to help in achieving the best LV lead position during CRT implantation. The authors were able to position the LV lead in a late activated myocardium and outside the scar area in 11 of the 14 patients. The 3 remaining patients had a scar area unavoidable due to anatomic limitations [29].

Strik et al. studied 67 CRT candidates with ECGi, wherein the intrinsic ADV amplitude accurately predicted the acute (AUC = 0.93) and chronic (AUC = 0.90) response to CRT. The amount of change in ADV by CRT only moderately predicted the responses (highest AUC = 0.76). A combined ECGi and acute hemodynamic study was performed in 26 patients with a median of 4 tested LV pacing sites. It was found that the change in LVdP/dtmax varied more between patients (−16% to +35%) than within patients (average of 11%), indicating the dominant effect of patient selection. Averaging LVdP/dtmax increases for all 104 LV pacing sites per LV segment (according to the American Heart Association) clearly demonstrated that, on a population level, basal and mid-lateral sites performed very well [20].

In line with these results, ECGi during LV optimization did not predict the LV pacing site with an optimal acute hemodynamic response. For example, the LV pacing site which resulted in the shortest ADV (51 ± 17 ms; most effective electrical resynchronization) did not result in greater increase in LVdP/dtmax when compared to the conventional basolateral LV pacing site (10 ± 13% versus 11 ± 14%, respectively, *p* = NS) [20,30].

ECGI combined with an anatomical imaging of the coronary venous root can help in guiding lead placement in CRT; however, the extent to which modification of the electrical substrate via LV pacing site optimization may only play a marginal role. This hypothesis is further supported by the computer modelling study conducted by Huntjens et al. [31], which elegantly shows that intrinsic interventricular dyssynchrony (as opposed to intra-LV dyssynchrony) is the dominant component of the electrical substrate driving the response to CRT and that dyssynchrony during BVP plays a minor role in this respect [32]. However, these conclusions should be counterbalanced with the relative low number of patients (n = 50), the absence of data on septal activation (epicardial map only) and the data exclusively collected during BiV pacing that cannot be translated to LBBAP or fusion CRT.

## 6. ECG Belt

ECG belt parameters were compared to ECGi; electrical parameters were recorded with both tools during intrinsic rhythm and BVP in 77 patients. There was a strong positive correlation between ECGi-LVAT and ECG belt-LTAT (R = 0.88; *p* < 0.001). There was also a strong correlation during BVP (R = 0.60; *p* = 0.01). There was also a strong correlation for ECGi-VEU and ECG belt SDAT during intrinsic rhythm (R = 0.76; *p* < 0.001). During BVP, there was only a weak correlation, which was not statistically significant (R = 0.29; *p* = 0.26). Finally, cranial or caudal displacement of the ECG belt did not affect either SDAT (*p* = 0.99) or LTAT (*p* = 0.99). Therefore, the ECG belt offers comparable measurements as ECGi, with significant cost advantages [33]. However, a lack of an anatomical landmark could be a significant limitation, especially for the optimal placement of an LV lead [28]. 

The diagnostic accuracy of the ECG belt to predict changes in LV end-systolic volume and LV ejection fraction after 6 months of CRT was tested in 66 candidates with CRT [34]. No sensitivity or specificity analysis was reported; however, patients with SDAT ≥ 35 ms had a greater improvement in EF (13 ± 8 vs. 4 ± 9 units, *p* < 0.001) and LVESV (−34 ± 28 vs. −13 ± 29%, *p* = 0.005). 

However, a multicenter, prospective, randomized, investigational research study was conducted at 48 centers in the United States, Canada and Europe on 408 subjects to test the hypothesis that, in patients traditionally less likely to respond to CRT, an individualized approach utilizing the ECG belt to guide lead placement, vector selection and device programming is superior to the current standard of care. The primary end point was based on a reduction in left ventricular end-systolic volume. The main result showed that while patients with higher SDAT at baseline had greater left ventricular reverse remodeling, an improvement in electrical dyssynchrony did not correlate with the extent of reverse remodeling [35]. This result again emphasizes the point that this response is mainly driven by baseline dyssynchrony rather than electrical optimization.

## 7. Limitations and Perspectives

There are main limitations to a widespread clinical application of ECGi, mostly because the existing techniques are costly, time-consuming and require prior radiation imaging. ECGi maps reveal highly homogeneous patterns of activation in patients with LBBB. Even if non-invasive cardiac mapping provides a more detailed depiction of the electrical substrate and helps in the understanding of the determinants of the CRT response, it would be difficult to demonstrate potential additional benefit from non-invasive mapping in patients with LBBB. Conversely, patients with non-ischemic cardiomyopathy (NICD) exhibit greater diversity and variability in activation patterns, likely explaining their higher non-response to CRT. There is potential for improvement in this subgroup by implementing personalized mapping and tailored therapy. Given that a significant portion of the clinical response is determined before implantation, primarily driven by the intrinsic electrical characteristics (with a high probability of success in patients with LBBB and lower probability in other ECG patterns), the impact of optimizing pacing site during the implantation procedure is expected to be relatively modest. This limits the practical utility of this technique during the implantation procedure. Moreover, the positioning of LV lead stimulation is frequently guided by the anatomical characteristics of the coronary venous system. Therefore, in practice, the site of stimulation cannot always match the latest area activated [29].

**Figure 4:**
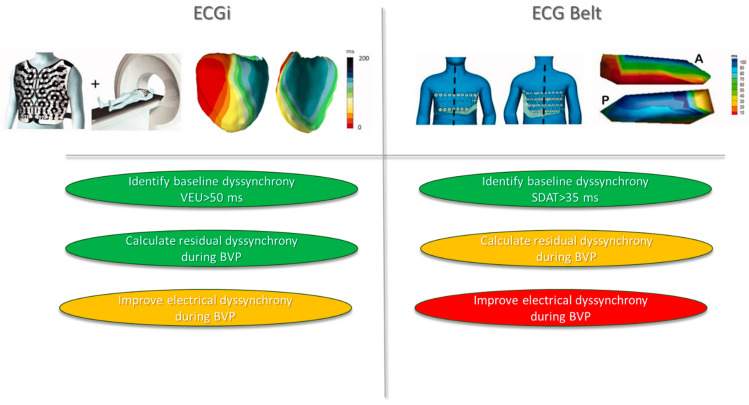
Role of ECGi and ECG belt in patient selection/optimization for CRT. Both the ECGi and ECG belt are validated tools to identify interventricular dyssynchrony, which is the main driver for CRT response. ECGi is a validated tool to improve understanding of biventricular activation during CRT. Both tools are inefficient to optimize CRT response; further study regarding whether ECGi can improve LV lead placement is warranted. ECGi: electrocardiographic imaging; CRT: cardiac resynchronization therapy.

The ECG belt does not require a scanner, making it easier to use at the cost of reduced geometric resolution. However, the ECG belt failed to demonstrate any clinical interest for BVP optimization in a recent multicenter study (Figure 4) [35]. 

In recent years, studies have identified new predictive markers of response: VEU, SSI, myocardial fibrosis rate and RV function. The VEU based solely on electrical activation measured via electrocardiographic mapping [17,31], and SSI based on a mechanical approach measured via speckle tracking [36,37], allow us to quantify ventricular asynchrony that is amendable with CRT. There is a threshold effect for the degree of asynchrony beyond which a response is highly probable [38].

Focal myocardial fibrosis measured on late-time MRI, as well as RV pump function, appear to play a role in the response to CRT [39,40]. The respective weights of these different parameters in determining a response are poorly understood. In addition, VEU and SSI are parameters of ventricular asynchrony, one electrical, the other mechanical, whose predictive power is not known to be equivalent or complementary. In fact, fibrosis and RV function appear to be the markers of choice, beyond the exploration of electrical or mechanical asynchrony, for optimizing response prediction, since they integrate different pathophysiological phenomena.

**Figure 5:**
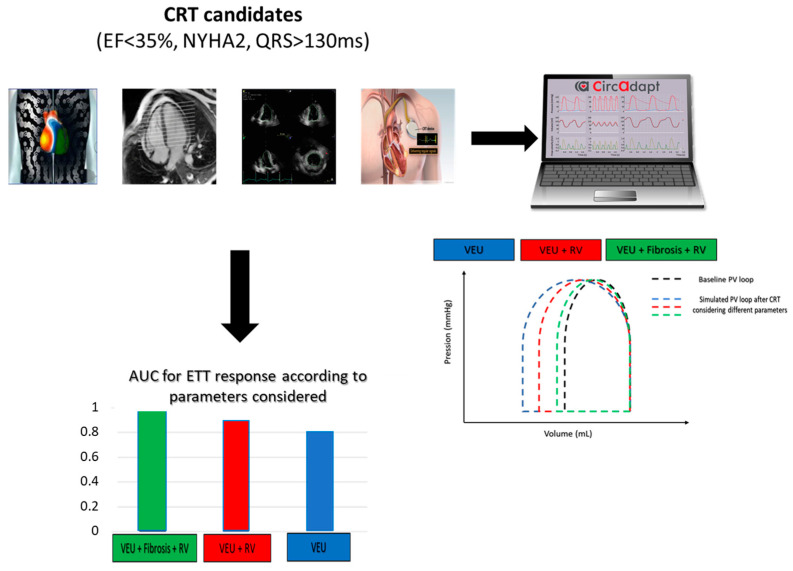
Theoretical multiparametric approach using a biophysical model (Circadapt Model) to predict response to cardiac resynchronization. For all patients undergoing CRT: measurement of VEU, SSI, fibrosis and RV function. Simulated flow variation values are obtained by considering one or more variables and obtaining an AUC for each simulation level. AUC values are given as examples. AUC: area under the curve; CRT: cardiac resynchronization therapy; RV: right ventricular; SSI: systolic stretch index; VEU: ventricular electrical uncoupling.

With the advent of artificial intelligence and increasingly powerful computer models, the future of cardiac resynchronization may lie in model-based patient selection. For example, the CircAdapt model allows the right and left ventricles to be subdivided into several segments, so that local electromechanical and interventricular inhomogeneity can be simulated [41]. Thus, segment activation times, as well as the electrical activation delay between the RV and LV (and therefore the VEU), are adjustable variables in CircAdapt, as are local or regional myocardial viability/rigidity and RV function. Variables such as VEU, SSI, fibrosis and RV function will be entered individually and jointly to explore the impact of each parameter on the variation in myocardial output in response to CRT. Thus, for each variable entered into the model, CircAdapt results in an individualized flow variation. These flow variations could be used to construct ROC curves (response prediction), enabling models to be compared with each other. This strategy is based on the assumption that acute variations in cardiac output are correlated with ventricular remodeling after CRT (Figure 5) [42].

## 8. Conclusions

The ECGi and ECG belt are efficient tools in patient selection for CRT through the identification of interventricular electrical dyssynchrony, which is the dominant driver of the CRT response. The target population comprises patients with an NICD and QRS duration of >130 ms. Future research needs to be conducted to explore the modulating effect of structural parameters, such as fibrosis or cardiac contractility. 

## Data Availability

No new data were created or analyzed in this study. Data sharing is not applicable to this article.

## Abbreviations

**ADV**	Activation delay vector
**BVP**	Biventricular pacing
**CRT**	Cardiac resynchronization therapy
**ECGi**	Electrocardiographic imaging
**HFrEF**	Heart failure with reduced ejection fraction
**LBBB**	Left bundle branch block
**LV**	Left ventricle
**LTAT**	Left thorax activation times
**NICD**	Non-specific intraventricular conduction delay
**RV**	Right ventricle
**RVAP**	Right ventricular apical pacing
**SDAT**	Standard deviation of activation times
**SSI**	Systolic stretch index
**TAT**	Total activation time
**VEU**	Ventricular electrical uncoupling
